# Rare case of postherpetic abdominal pseudohernia in a patient on peritoneal dialysis

**DOI:** 10.1007/s40620-024-01954-3

**Published:** 2024-06-05

**Authors:** Agnieszka Przygocka, Giacomo Magnoni, Matilde Picotti, Renato Rapanà, Gaetano La Manna

**Affiliations:** 1 Nephrology and Dialysis Unit, Santa Maria della Scaletta Hospital, Imola, Italy; 2 Nephrology, Dialysis and Kidney Transplant Unit, IRCCS Azienda Ospedaliero-Universitaria di Bologna, Bologna, Italy; 3 Department of Medical and Surgical Sciences (DIMEC), Alma Mater Studiorum - University of Bologna, Bologna, Italy

**Keywords:** Postherpetic abdominal hernia, Herpes zoster infection, Peritoneal dialysis, End-stage renal disease

## Abstract

Patients affected by chronic kidney disease, especially those requiring maintenance dialysis therapy, are particularly susceptible to infections, including reactivation of herpes zoster and are also at increased risk of herpes zoster complications. Postherpetic abdominal pseudohernia is a rare sequela of the infection, caused by motor neuropathy with muscle paresis, that manifests as an abdominal protrusion. In patients receiving peritoneal dialysis who may often present slight abdominal distension, the diagnosis of this complication may be challenging. We present a case of this rare neurological complication in a patient on peritoneal dialysis and discuss its etiology and management. To the best of our knowledge, this is the first report of postherpetic abdominal pseudohernia in a patient receiving kidney replacement therapy.

## Introduction

Postherpetic abdominal pseudohernia is a highly uncommon complication of herpes zoster infection, that is due to varicella zoster virus reactivation. It is caused by paresis of the ipsilateral abdominal muscles following herpes zoster infection and manifests as an abdominal bulge. The diagnosis is clinical and is based on its temporal association with herpes zoster, as it may occur one to eight weeks after the appearance of a herpetic rash [1, 2]. We report a case of abdominal pseudohernia due to herpes zoster in a patient on peritoneal dialysis (PD).

## Case report

A 40-year-old Caucasian woman affected by end-stage kidney disease (ESKD) of unknown etiology, on automated PD, presented intense left flank pain followed by the onset of an erythematous rash and blisters. She had a history of arterial hypertension, kidney transplant complicated with graft failure due to chronic rejection 3 years after transplantation, and melanoma of the lower limb treated with local excision surgery, currently in remission. Her pharmacological therapy included low-dose prednisone (2.5 mg/day). Given the clinical presentation, the diagnosis of herpes zoster infection was made and she was treated with oral acyclovir therapy for 14 days. On day 16, a left abdominal protrusion with local paresthesia and hyperalgesia was noted; the results of the remaining neurological examination were normal. Unenhanced abdominal computed tomography was performed, ruling out abdominal masses and true abdominal hernia; diffuse coprostasis was observed without significant bowel distension. Thus, she was diagnosed with postherpetic abdominal pseudohernia and treated with oral gabapentin for the neuropathic pain, which improved her symptoms. Subsequently, neurological evaluation was performed; given the good response to treatment, electromyography was not indicated and gabapentin therapy was continued for one month. Three months after the initial presentation complete resolution of symptoms was observed.

## Discussion

After a primary infection caused by varicella zoster virus, the virus remains latent in the cranial nerve ganglia and dorsal root ganglia of the spinal cord [3]. Older and immunocompromised individuals are at higher risk of disease reactivation. Patients affected by chronic kidney disease, especially those requiring maintenance dialysis therapy, are particularly susceptible to infections, including reactivation of varicella zoster virus, as uremia is associated with impaired lymphocyte B and T function [4–6]. Available data suggest that individuals with underlying comorbidities such as impaired kidney function, in particular kidney transplant recipients and patients on PD, are also at increased risk of herpes zoster complications [7]. A number of complications may occur, with neurological and ocular sequelae resulting in notable challenges. Across the neurological spectrum, postherpetic neuralgia is the most common. Varicella zoster virus can also cause meningoencephalitis, meningoradiculitis, cerebellitis and myelopathy [3].

Postherpetic pseudohernia is an uncommon neurological complication of herpes zoster infection characterized by motor involvement that leads to muscle paresis and abdominal protrusion [1]. The true incidence is difficult to establish and has been reported to be between 0 and 5% in different studies [8–10]. The most frequently involved dermatome is T11 [1, 2]. It usually occurs one to eight weeks after the onset of herpetic rash but in rare cases may appear before or without cutaneous eruption [1, 2, 11]. The diagnosis is based on clinical presentation and temporal correlation with herpes zoster infection as well as exclusion of other causes. Therefore, radiological imaging such as ultrasonography, computed tomography or magnetic resonance should be performed to exclude true abdominal hernias and intra-abdominal masses [2]. Electrophysiological studies are not routinely performed but they can be useful for evaluating the extent of muscle paresis or for clearing up diagnostic doubts. In patients with abdominal pseudohernia who underwent electromyography, denervation features were detected in 95–100% of cases [1, 2].

This disorder can be associated with visceral neuropathy affecting the gastrointestinal tract, which most commonly manifests as constipation. Such complication was reported in 19.4% of patients in the analysis by Chernev et al*.* [1] and can be particularly problematic in the PD population. Given the computed tomography findings, this gastrointestinal disorder was suspected in our patient, and she was treated accordingly with increased osmotic laxative therapy.

Treatment is conservative, and in most of the documented cases, the symptoms resolve without specific therapy. Based on the clinical presentation, the use of a corset and pain management can be prescribed; in patients with neuropathic pain, gabapentin is helpful, as is the case for postherpetic neuralgia [3, 11, 12]. The prognosis of postherpetic pseudohernia appears to be good. Chernev et al*.* reported that 79.3% of patients achieved complete recovery in one year, with a mean time to recovery of 4.9 months, whereas Chiew et al. documented full resolution of abdominal paresis in 70% of patients, with a median follow-up of 3 months [1, 2].

**Fig. 1:**
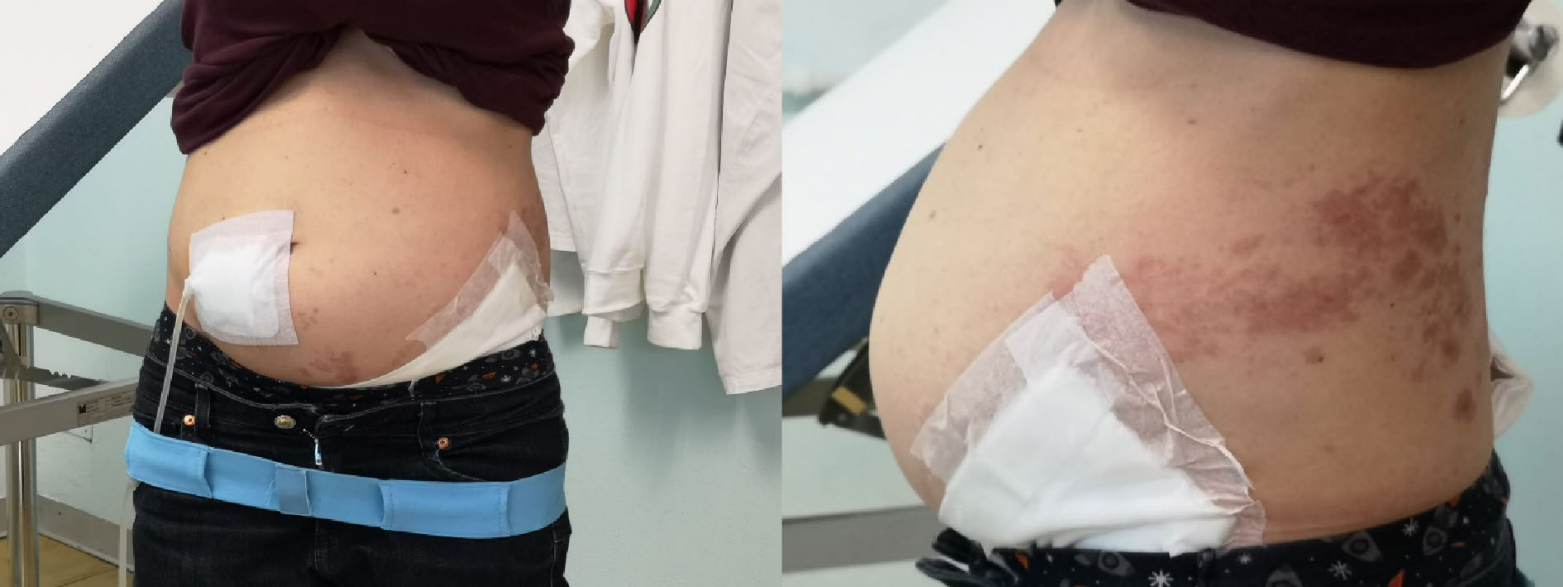
The left abdominal wall bulge was more prominent when the patient was in the standing position. Herpetic cutaneous lesion is visible on the left flank

**Fig. 2:**
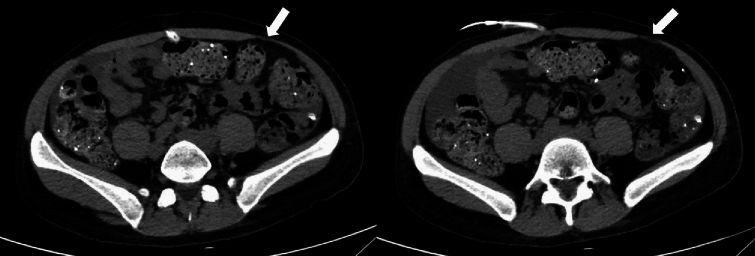
Abdominal CT demonstrated thinning of the left external oblique and internal oblique muscles (arrows). Abdominal masses and true abdominal hernias were excluded

To the best of our knowledge, this is the first report of postherpetic abdominal pseudohernia in a patient receiving kidney replacement therapy. Given the slight chronic abdominal distension present in patients on PD, the clinical manifestation of this disorder may not be as evident in this group. However, it should be considered in case of abdominal asymmetry after herpes zoster infection. Moreover, our experience suggests that the treatment previously reported to be successful in the general population, was efficient and well tolerated in a patient affected by ESKD, after adjusting the dose for kidney function. Raising awareness of this rare complication may save costly diagnostic investigations and improve the management of possible sequelae of herpes zoster (Figs. 1 and 2).

## Data Availability

The data generated and analyzed in this case are presented within the manuscript.

## Abbreviations

ESRD: End-stage renal disease
PD: Peritoneal dialysis
